# Peer Review of “Interpreting the Estimand Framework From a Causal Inference Perspective”

**DOI:** 10.2196/98122

**Published:** 2026-05-22

**Authors:** Linying Zhang

**Affiliations:** 1 Washington University in St Louis, St. Louis, MO, United States

**Keywords:** causal inference, clinical trial, estimand, intercurrent event, treatment effect

*This is a peer review report for “Interpreting the Estimand Framework From a Causal Inference Perspective.”*

## Round 1 Review

This Viewpoint [1] uses mathematical expressions to formalize the estimand framework proposed by a professional society (ie, the ICH), targeting a pharmaceutical industry audience involved in clinical trial design and analysis. While the mathematical formulations for the estimation strategies appear technically correct, the article lacks clarity regarding the significance of the problem it aims to address. The overall discussion remains superficial, offering limited conceptual or practical contributions to the existing literature.

### Major Comments

1. The professional society “ICH” is never spelled out or introduced. Additional context is needed regarding the role of the ICH, its influence on regulatory science, and why its guidelines are particularly important for clinical trial design and analysis.
2. The Efficacy Guideline E9 was published in 2019. The authors should clarify what impact this guideline has had on the pharmaceutical industry since its release. Moreover, it is unclear why a causal interpretation of this guideline is timely and important in 2025, several years after its publication.
3. None of the proposed strategies address noncompliance, such as cases where treatment is not received despite assignment or is received without assignment (eg, X(*R*=1)=0 or X(*R*=0)=1). Noncompliance is a central issue in causal inference and should be explicitly discussed. If noncompliance is assumed to be irrelevant, then the introduction of the notation R appears redundant and should be justified or removed.
4. The strategies are presented at a very high level. Although the 4 cases illustrated in Figure 2 provide some intuition regarding the appropriateness of each strategy, the Viewpoint would be substantially strengthened by grounding the discussion in real clinical trial examples. Demonstrating how each strategy has been applied in practice would greatly improve clarity and impact.
5. The scope and framing of the Viewpoint appear better suited for a pharmaceutical science or regulatory-focused journal rather than a JMIR-based journal. The authors should better justify the relevance of this work to the JMIR readership or reconsider the target venue.

### Minor Comments

1. Section 2 begins with the statement: “A causal inference framework is based on the potential outcome framework.” This is inaccurate, as causal inference can also be grounded in other frameworks, such as structural causal models.
2. In the abstract, the sentence “This article aims to interpret the estimand framework through its underlying theories, the causal inference framework based on potential outcomes” should replace the comma with “and” for grammatical correctness.
3. On page 2, second line: “Generally, Treaments are…” — the “T” in “Treatments” should not be capitalized.
4. In section 3.2 (page 6): the sentence “Through the hypothetical strategy, we make the second…” is ambiguous and should be rewritten for clarity.

## Round 2 Review

I appreciate the authors taking the time to address the reviewers’ comments. I have read the revised manuscript, but I still find that the Viewpoint is too incremental and not a strong fit for the journal’s audience. It is not sufficiently well motivated why translating this particular guideline into the potential outcomes framework is important for researchers across settings, from clinical trials to observational studies. While the potential outcomes framework is already widely used, it remains unclear why aligning it specifically with this guideline represents a meaningful or novel contribution that warrants a Viewpoint article.
